# Topographical and Chronological Analysis of Thin Cutaneous Melanoma’s Progressions: A Multicentric Study

**DOI:** 10.3390/cancers15153989

**Published:** 2023-08-06

**Authors:** Emmanouil Chousakos, Daniela Zugna, Emi Dika, Aram Boada, Sebastian Podlipnik, Cristina Carrera, Josep Malvehy, Susana Puig, Celia Requena, Esperanza Manrique-Silva, Eduardo Nagore, Pietro Quaglino, Rebecca Senetta, Simone Ribero

**Affiliations:** 1 1st Department of Pathology, Medical School, National and Kapodistrian University of Athens, 11527 Athens, Greece; 2 Cancer Epidemiology Unit, Department of Medical Sciences, University of Turin, 10126 Turin, Italy; daniela.zugna@unito.it; 3 Oncologic Dermatology Unit, IRCCS Azienda Ospedaliero-Universitaria di Bologna, 40138 Bologna, Italy; emi.dika3@unibo.it; 4 Department of Medical and Surgical Sciences (DIMEC), University of Bologna, 40138 Bologna, Italy; 5 Dermatology Department, Hospital Universitari Germans Trias i Pujol, Institut d’Investigació Germans Trias i Pujol, 08916 Badalona, Spain; aramboada@gmail.com; 6 Melanoma Unit, Dermatology Department, Hospital Clinic, Universitat de Barcelona, Institut d’ Investigacions Biomèdiques August Pi i Sunyer (IDIBAPS), 08036 Barcelona, Spain; spodlipnik@gmail.com (S.P.); criscarrer@yahoo.es (C.C.); jmalvehy@gmail.com (J.M.); susipuig@gmail.com (S.P.); 7 CIBER de Enfermedades Raras, Instituto de Salud Carlos III, 28029 Barcelona, Spain; 8 Dermatology Department, Instituto Valenciano de Oncología, 46009 Valencia, Spain; celiareq@hotmail.com (C.R.); emanriques19@gmail.com (E.M.-S.); eduardo.nagore.e@gmail.com (E.N.); 9 Dermatology Clinic, Medical Sciences Department, University of Turin, 10126 Turin, Italy; pietro.quaglino@unito.it (P.Q.); simone.ribero@unito.it (S.R.); 10 Pathology Unit, Department of Oncology, University of Turin, 10124 Turin, Italy; rebesenetta@gmail.com

**Keywords:** cutaneous melanoma, invasive melanoma, metastatic melanoma, thin melanoma, melanoma progressions, thin melanoma natural history, thin melanoma metastasis, thin melanoma progression, metastatic pattern, progression pattern

## Abstract

**Simple Summary:**

A big part of the increased incidence of melanoma can be attributed to thin, cutaneous melanomas (≤1 mm Breslow thickness), which can rarely progress. The aim of this study is to analyze the sequences of localizations and time intervals of thin melanomas’ progressions and investigate their associations with clinicopathological features of the primary and metastatic tumors. We collected 204 eligible cases from five specialized centers across Europe. The first progressions occurred locally (24%), in regional lymph nodes (15%), and in distant sites (61%) after a median time of 3.10 years. The median elapsed time between first and second progression and between second and third was 0.82 and 0.49 years, respectively, while the median survival time was about 4 years since first progression. Our findings describe the natural history of thin melanoma and dictate optimized management and follow-up, especially for subgroups at a higher risk for metastasis.

**Abstract:**

A great portion of cutaneous melanoma’s diagnoses nowadays is attributed to thin tumors with up to 1 mm in Breslow thickness (hereafter thin CMs), which occasionally metastasize. The objective of this study was to identify thin CM’s metastatic patterns from a topographical and chronological standpoint. A total of 204 cases of metastatic thin CMs from five specialized centers were included in the study, and corresponding data were collected (clinical, epidemiological, histopathological information of primary tumor and the number, anatomical site, and time intervals of their progressions). First progressions occurred locally, in regional lymph nodes, and in a distant site in 24%, 15% and 61% of cases, respectively, with a median time to first progression of 3.10 years (IQR: 1.09–5.24). The median elapsed time between the first and second progression and between the second and third progression was 0.82 (IQR: 0.34–1.97) and 0.49 (IQR: 0.21–2.30) years, respectively, while the median survival time was about 4 years since first progression. Furthermore, the sequences of locations and time intervals of the progressions were associated with the clinicopathological and demographic features of the primary tumors along with the features of the preceding progressions. In conclusion, the findings of this study describe the natural history of thin CMs, thus highlighting the necessity to identify subgroups of thin CMs at a higher risk for metastasis and contributing to the optimization of the management and follow-up of thin CM patients.

## 1. Introduction

The gradually improving comprehension of the oncogenic pathways of cutaneous melanoma (CM) and the mechanisms of its progression, as well as the optimization of preventive, diagnostic and follow-up modalities and protocols, have facilitated diagnosis at earlier stages. However, the overall melanoma mortality has not improved significantly [1,2]. It is evident that the prognosis of CM patients is dramatically impacted by the tumor’s profile with respect to its metastatic potential at the time of diagnosis [3,4]. Therefore, there has been a widespread interest regarding the pathways of CM metastasis, with recent studies supporting a simultaneous, parallel pathway in contrast to the long-standing prevailing theory of a serial pattern of metastasis [5,6,7]. The incidence of melanoma has increased steadily during the last decades, but this is mostly because of the noticeable increase of thin CMs (≤1 mm), and, therefore, a high proportion of melanoma-related deaths derive from them, despite their lower mortality rates compared to the thicker ones [8,9].

Thin CMs consist of invasive CMs of any histological subtype with a Breslow thickness of up to 1 mm, which are thus classified as stage T1a or T1b according to the latest 8th AJCC staging [10]. Even though they generally manifest a lower metastatic potential, some of them can rarely progress to regional and/or distant sites. It has been recently reported in south European populations that 10.23% of metastatic melanoma patients were initially diagnosed with stage Ia tumors [11]. Many studies have aimed to identify the association between certain molecular, histopathological, and clinical characteristics of thin CMs with a higher risk for progression [7,12], as well as with the time course of thin CM progression incidences [13]. Nevertheless, the natural history of the entity is not clearly defined. Furthermore, the performance of sentinel lymph node biopsy (SLNB) in this specific subgroup of CMs is still a matter of controversy among experts [14,15] in the context of the absence of strong recommendations in the latest guidelines for the management of stage 1 CMs [16,17].

The aim of this multicenter study was to analyze the sequence of events during the monitoring course of metastatic, thin CMs from a topographical and a timeframe standpoint in an effort to identify and further understand their patterns of progression and natural history. Such an analysis could provide insights regarding the underlying metastatic pathways of thin CMs, as well as modify our surveillance algorithms and optimize our methodology for the earlier detection of a thin CM’s progression.

## 2. Materials and Methods

### 2.1. Data

The retrospective observational study was based on the clinical data from 207 cases of thin CMs that progressed. These data were retrospectively collected from the archives of five participating melanoma units: Barcelona (Hospital Clinic), Badalona (Hospital Universitari Germans Trials i Pujol), Valencia (Instituto Valenciano de Oncologia), Bologna (Sant’Orsola-Malpighi Hospital), and Turin (Turin University Hospital “Città della Salute e della Scienza”). Thin CMs were defined as those with a Breslow thickness less than or equal to 1 mm. Patients with a thin CM were eligible if they had manifested at least one recorded progression, regardless of its localization, and for which specific epidemiological, histopathological, and disease course-related data were recorded. The follow-up protocol was similar among all centers, as per the standard practices and guidelines. Data on the clinical and histopathological features of the primary CM, the date and localization of the first, second, or third progression, if it existed, the patient’s life status, the melanoma-specific mortality, and the date of death or latest follow-up were collected from every patient. With the exception of Bologna, which recorded up to two progressions, all other centers recorded up to three progressions. All centers followed up with patients until their last clinical visit or death. The Bologna database did not record any third progression, but they reported data until a second one; thus, this cohort was not included in the analysis of the third progression. Of note is the fact that only five thin CM patients were diagnosed with a first progression after 2018; therefore, most of our cohort did not receive adjuvant therapies.

### 2.2. Statistical Analysis

Patients and their progressions were analyzed, and odds ratios of the type of the first progression according to patients’ and tumors’ characteristics were estimated by multinomial logistic regression. Three outcomes of interest were modeled: (i) the second progression, (ii) the third progression—conditional on having a second progression—and (iii) the overall melanoma-specific survival. When modeling the risk of second progression, death from any cause was considered a competing risk, with the time since the first progression used as the principal timescale. When modeling the risk of third progression, death from any cause was considered a competing risk, with the time since the second progression used as the principal timescale. When modeling the melanoma-specific mortality, death from causes different from melanoma was considered a competing risk, with the time since the first progression used as the principal timescale and the second and third progressions used as time-dependent variables.

We evaluated the impact of several variables on the progression of thin CMs: sex and age at diagnosis, the anatomic site of the primary tumor classified in four categories (head or neck, trunk, arms, and legs), the histological subtypes of the CM [classified as lentigo maligna melanoma (LMM), superficial-spreading melanoma (SSM), nodular melanoma (NM), and acral lentiginous melanoma (ALM)], the Breslow thickness, the presence or lack thereof of ulceration, the elapsed time between the diagnosis, and the progression and type of progression classified as local (local, in transit, satellite, cutaneous), nodal, or distant (subcutaneous and other organs).

Specifically, when modeling the risk of the second progression, the age at first progression, the elapsed time between diagnosis, and the first progression and type of first progression were included into the model. When modeling the risk of the third progression, the age at second progression, the elapsed time between diagnosis and second progression, and the type of first and second progression were included into the model. When modeling the melanoma-specific mortality, the age at diagnosis, the elapsed time between diagnosis and first progression, and the type of first progression were included into the model, and the second and the third progressions were considered as time-dependent variables.

Nonparametric, cause-specific cumulative incidence functions were calculated for all three outcomes of interest. The outcome-specific cumulative incidence curve represents the probability of that outcome occurring before other competing events on the specific timescale. 

The association between the individual, clinicopathological, and time-course characteristics was estimated by a multivariable competing risks proportional subdistribution hazards model. This model allows for the estimation of the hazard corresponding to the cause-specific cumulative incidence function in the presence of randomly right-censored and left-truncated data. Specifically, it leads to estimates of the subdistribution hazard ratio (sub-HR). To deal with nonlinear trends, the age, elapsed time, and Breslow score were modeled by restricted cubic splines. Models, including linear and nonlinear trends, were compared using the likelihood ratio test. Hence, the elapsed time was further categorized in 4 categories (less than 1 year, 1–2 years, 2–3 years, and more than 3 years) in order to make the effect estimates more interpretable. We checked and tested whether the effect of the variables included in the model was proportional using Schoenfeld’s residuals.

The main analyses concerning the risk of third progression and the melanoma-specific mortality risk were conducted, with the exclusion of the Bologna center because of missing data on the third progression. However, the secondary analysis on the melanoma-specific mortality risk also included the Bologna center, without considering the third progression as a time-dependent variable in the model.

## 3. Results

### 3.1. Study Population

**Figure 1:**
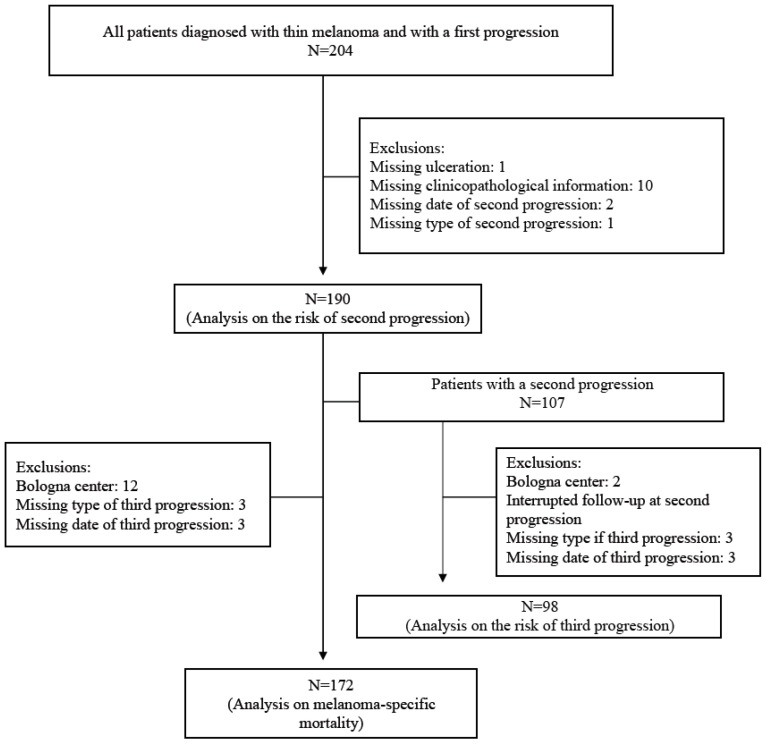
Flowchart of the patients’ selection.

A flow chart of patients’ selection is shown in Figure 1. Of the 207 study participants, 3 patients without information after the first progression were excluded. Analyses were, therefore, based on 204 patients, the characteristics of whom are described in Table 1. The proportion of males and females was similar (49% vs. 51%) and the median age at diagnosis was 53 years (interquartile range: IQR: 40–65). The most common anatomic site of the primary tumor was the trunk (47%), followed by the legs (27%) and the arms/head and neck (13%); the most common histotype of CM was the SSM (80%), followed by the LMM (7%), NM (7%), and ALM (6%). The median Breslow thickness of the neoplasms was 0.8 mm (IQR: 0.6–0.9). The study’s thin CM cases were diagnosed between 1975 and 2018. The last controls of the sample’s patients, namely, the latest follow-up or occurrence of death, regardless of cause, covered the calendar period of 1977–2021 (median: 2012, IQR: 2003–2018).

### 3.2. Progression Pathways

**Figure 2:**
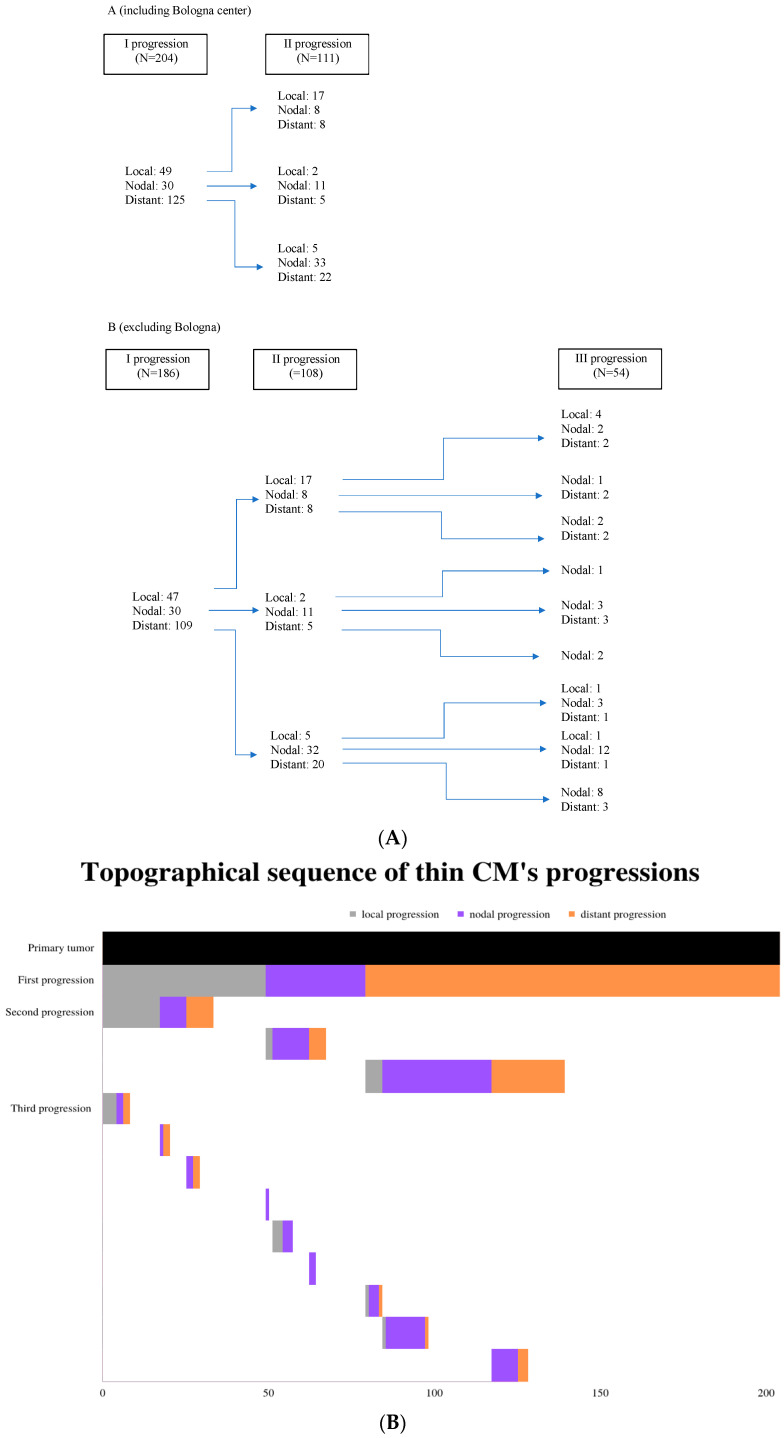
Progression pathways. (**A**) Panel A includes Bologna center and, hence, data on first and second progressions. Panel B excludes Bologna center cases. (**B**) Schematic presentation of the topographical sequences of progressions for the entire sample.

The median age of the patients at the first progression was 59 years (IQR: 45–69). The type of progression was initially divided in three categories according to the progression’s anatomical site with respect to the location of the initial tumor: (i) local, including the recurrence of the initial tumor and in transit or satellite metastasis; (ii) nodal; and (iii) distant. After the initial categorization of the progression, each case was further subcategorized based on the specific anatomical site of the progression. Nodal progressions that occurred in lymph nodes that were not draining the anatomical region of the primary tumor were classified as distant progressions, as the underlying mechanism of dissemination was hematogenous. The progression pathways are shown in Figure 2. The following analyses were conducted on complete data, because there were very few missing data.

### 3.3. First Progression

Of the first progressions, 24% occurred locally, 15% occurred in a lymph node, and 61% occurred in a distant site. The median elapsed time between the diagnosis and first progression was 3.10 years (IQR: 1.09–5.24). Using a multinomial logistic regression, the odds ratio for the local first progression compared to the distant first progression would be expected to increase by a factor of 4.22 (95% CI: 1.06,16.77) for head/neck compared to trunk and by a factor of 3.49 (95% CI: 1.30,9.39) for leg compared to trunk, and to decrease by a factor of 0.11 (95% CI: 0.02,0.72) for a unit increase in the Breslow score. The odds ratio for the nodal first progression compared to the distant first progression would be expected to increase by a factor of 2.77 (95% CI: 1.06,7.28) for females compared to males, by a factor of 9.88 (95% CI: 1.40,69.79) for ALM compared to SSM, by a factor of 1.05 (95% CI: 1.01,1.08) for a unit increase of age at diagnosis, and to decrease by a factor of 0.15 (95% CI: 0.03,0.83) for legs compared to the trunk.

### 3.4. Second Progression

**Figure 3:**
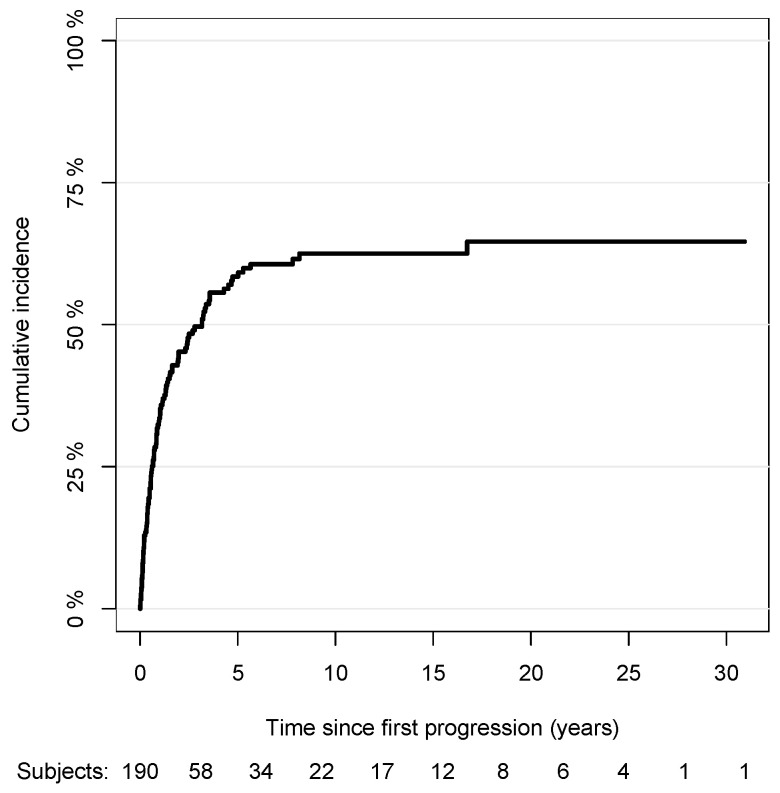
Nonparametric cumulative incidence of second progression with respect to time since first progression.

**Figure 4:**
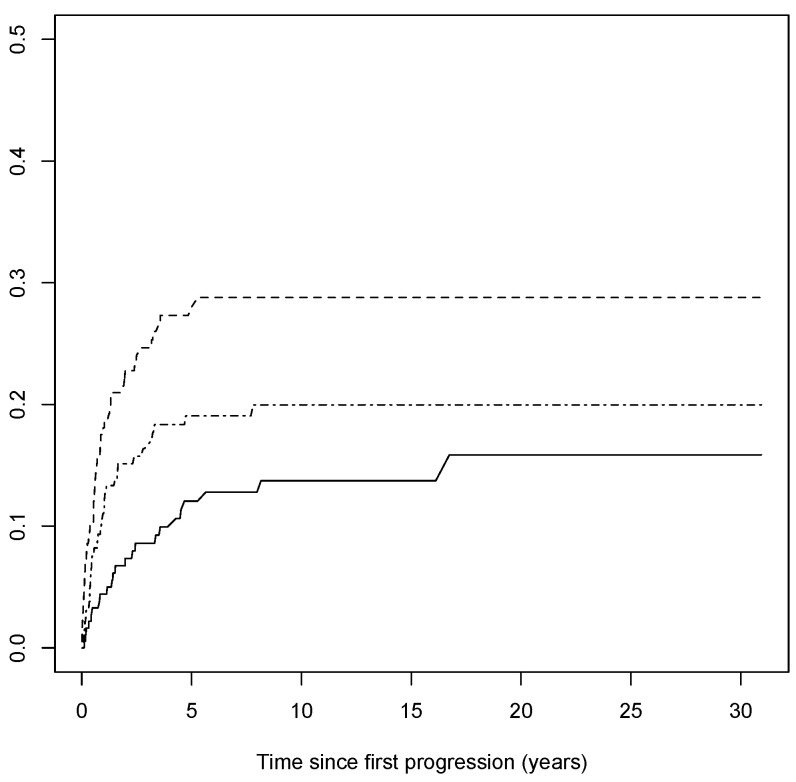
Nonparametric cumulative incidence of the type of second progression with respect to time since first progression. Solid line represents the subgroup of local progressions of thin CMs, dashed line represents the subgroup of nodal ones, and dash–dotted line represents the subgroup of distant ones.

Out of 190 patients with complete data, 107 patients had a second progression, and 25 died from any causes. Among patients with a second progression, the median elapsed time between the first and second progression was 0.82 years (IQR: 0.34–1.97). The cumulative incidence curve of the second progression with respect to the time since the first progression is shown in Figure 3 (the median survival time to second progression was 3.2 years since the first progression). Out of 107 progressions, 23 were local, 50 were nodal, and 34 were distant. The cumulative incidence curves of the local, nodal, and distant second progressions, having considered competing events, with respect to the time since the first progression are shown in Figure 4. The association between the risk of the second progression and the variables included in the multivariable model are reported in Table 2. The risk of the second progression was independent of gender (male vs. female: sub-HR: 0.83, 95% CI: 0.54,1.27), anatomic site of tumor (head/neck vs. trunk: sub-HR: 1.50, 95% CI: 0.69,3.26; arms: sub-HR: 0.94, 95% CI: 0.52,1.71; legs: sub-HR: 1.06, 95% CI: 0.63,1.68), Breslow score (unit increase, sub-HR: 2.13, 95% CI: 0.80,5.66), presence of ulceration (sub-HR: 1.48, 95% CI: 0.82,2.70), SLNB performed or not (sub-HR: 0.79, 95% CI: 0.50,1.25), and age at diagnosis (unit increase, sub-HR: 1.01, 95% CI: 0.99,1.02). Patients diagnosed with LMM were at a lower risk of second progression compared to patients diagnosed with SSM (sub-HR: 0.32, 95% CI: 0.14,0.76), while no difference in the estimated risk was observed for the other histopathological forms of cutaneous melanoma (NM vs. SSM: sub-HR: 1.02, 95% CI: 0.43,2.45; ALM vs. SSM: sub-HR: 0.65, 95% CI: 0.21,1.98). Being diagnosed with a distant first progression compared to a local first progression decreased the risk of a second progression (sub-HR: 0.67, 95% CI: 0.42,1.05), while no difference was observed for nodal first progressions (sub-HR: 0.85, 95% CI: 0.45,1.58). Although not having any statistical significance, the risk of a second progression increased over time since the first progression, more remarkably in the first 3 years, and then decreased slightly ([1,2) vs. [0,1) years: sub-HR: 1.26, 95% CI: 0.60,2.65; [2,3) vs. [0,1): sub-HR: 1.72, 95% CI: 0.83,3.53; 3+ vs. [0,1): sub-HR: 1.10, 95% CI: 0.62,1.95).

### 3.5. Third Progression

**Figure 5:**
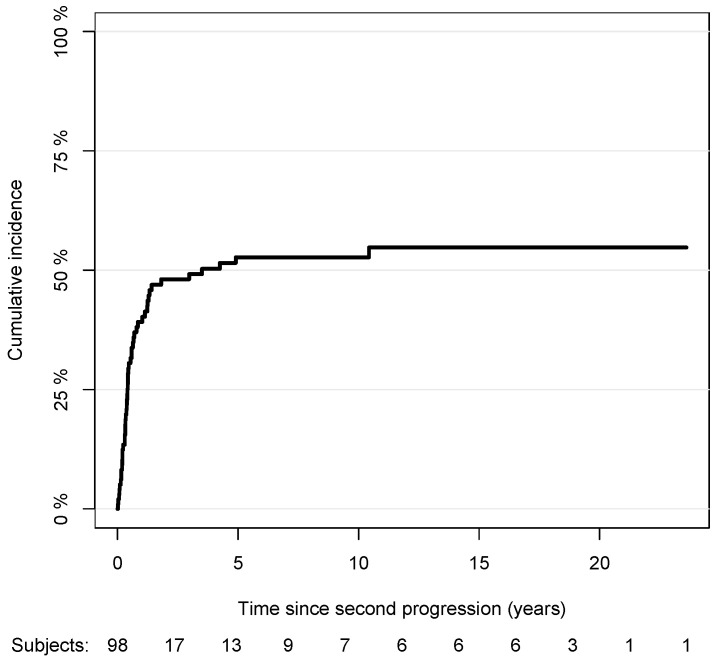
Nonparametric cumulative incidence of third progression with respect to time since second progression.

Out of 107 patients with a second progression, 1 patient with an interrupted follow-up at the second progression, 6 patients without data on the type or date of the third progression, and 2 patients from the Bologna center were excluded. Among 98 patients included in the analysis, 50 patients had a third progression, and 35 failed from a competing event, namely, from death of any cause. For patients with a third progression, the median elapsed time between the second and third progression was about 0.49 years (IQR: 0.21–2.30). The cumulative incidence curve of the third progression with respect to the time since the second progression is shown in Figure 5 (the median survival time to the third progression was 3.5 years since the second progression). The association between the risk of the third progression and the variables included in the multivariable model are reported in Table 3. The risk of the third progression was independent of gender (male vs. female: sub-HR: 1.04, 95% CI: 0.48,2.25), anatomic site of tumor (head/neck vs. trunk: sub-HR: 1.20, 95% CI: 0.41,3.48; arms: sub-HR: 0.47, 95% CI: 0.13,1.72; legs: sub-HR: 0.63, 95% CI: 0.27,1.47), Breslow score (unit increase, sub-HR: 0.46, 95% CI: 0.09,2.29), histopathological type of CM (LMM vs. SSM: sub-HR: 0.34, 95% CI: 0.09,1.33; NM vs. SSM: sub-HR: 1.80, 95% CI: 0.40,8.11, ALM vs. SSM: sub-HR: 1.44, 95% CI: 0.4,5.17), presence of ulceration (sub-HR: 0.32, 95% CI: 0.08,1.23), SLNB performance (sub-HR: 1.37, 95% CI: 0.67,2.81), age at diagnosis (unit increase, sub-HR: 0.98, 95% CI: 0.96,1.01), and elapsed time between the diagnosis and second progression ([2–4) vs. [0,2) years: sub-HR: 0.78, 95%, CI: 0.25,2.49; [4,6) vs. [0,1): sub-HR: 1.11, 95% CI: 0.31,4.03; 6+ vs. [0,2): sub-HR: 1.20, 95% CI: 0.36,3.98). No difference was observed in the risk for the third progression for patients with nodal and distant second progressions compared to those with local ones (sub-HR: 0.52, 95% CI: 0.23,1.18 and sub-HR: 1.09, 95% CI: 0.44,2.65, respectively).

**Figure 6:**
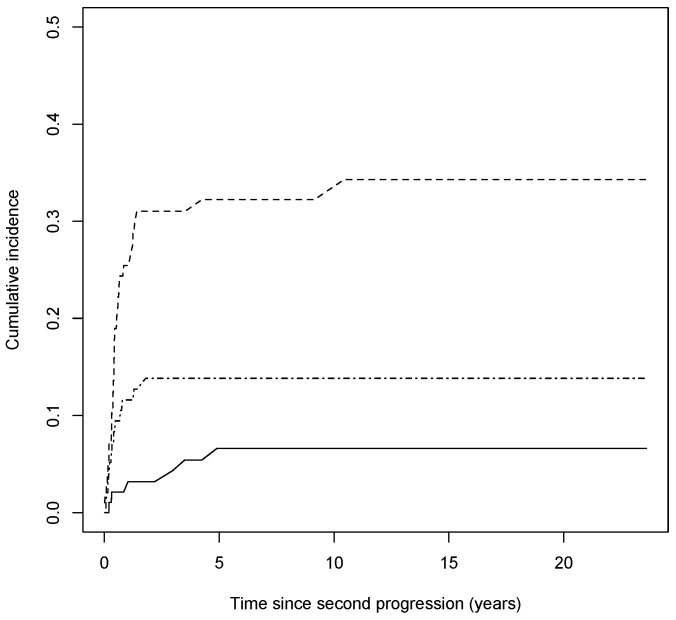
Nonparametric cumulative incidence of the type of third progression over time since second progression. Solid line represents the subgroup of local progressions of thin CMs, dashed line represents the subgroup of nodal ones, and dash–dotted line represents the subgroup of distant ones.

Out of 50 third progressions, 6 were local, 31 were nodal, and 13 were distant. The cumulative incidence curves of the local, nodal, and distant third progressions, having considered competing events, with respect to the time since the first progression are shown in Figure 6.

### 3.6. Melanoma-Specific Mortality

**Figure 7:**
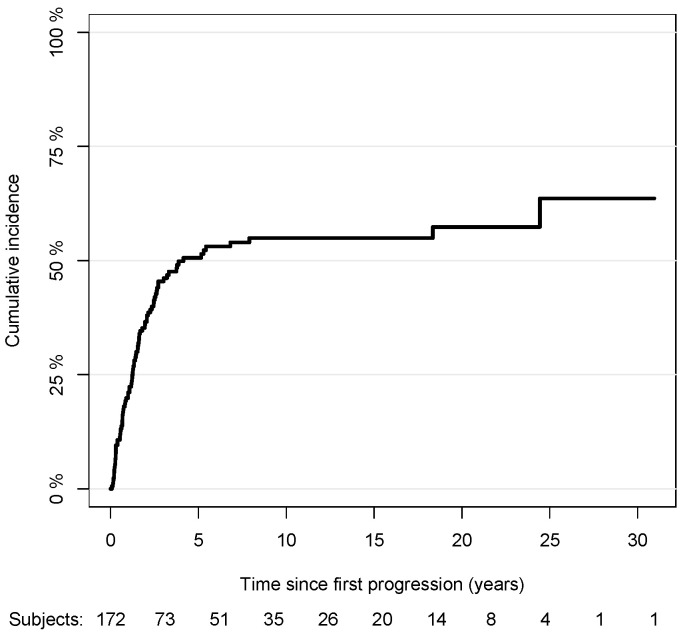
Nonparametric cumulative incidence of melanoma-specific mortality with respect to time since first progression.

Out of 172 patients with complete data on the progression pathways, 86 patients died from melanoma, and 9 died from other causes. The cumulative incidence curve of the melanoma-specific mortality with respect to the time since the first progression is shown in Figure 7. The median survival time was about 4 years since the first progression. The association between the risk of melanoma-specific mortality and the variables included in the multivariable model are reported in Table 4. The risk of death was independent of gender (male vs. female: sub-HR: 0.96, 95% CI: 0.58,1.60), anatomic site of tumor (head/neck vs. trunk: sub-HR: 1.11, 95% CI: 0.53,2.33; arms: sub-HR: 0.22, 95% CI: 0.23,1.21; legs: sub-HR: 0.59, 95% CI: 0.29,1.19), histopathological type of CM (LMM vs. SSM: sub-HR: 0.43, 95% CI: 0.09,2.08; NM vs. SSM: sub-HR: 1.11, 95% CI: 0.34,3.61, ALM vs. SSM: sub-HR: 2.18, 95% CI: 0.58,8.10), Breslow score (unit increase, sub-HR: 2.46, 95% CI: 0.60,10.10), presence of ulceration (sub-HR: 1.73, 95% CI: 0.79,3.85), performance of SLNB (sub-HR: 0.84, 95% CI: 0.42,1.67), age at first progression (sub-HR: 1.01, 95% CI: 0.99,1.03), and elapsed time between the diagnosis and first progression ([1,2) vs. [0,1) years: sub-HR: 1.29, 95% CI: 0.54,3.07; [2,3) vs. [0,1): sub-HR:1.35, 95% CI: 0.53,3.46; 3+ vs. [0,1): sub-HR: 1.34, 95% CI: 0.61,2.91). The occurrence of a nodal or distant first progression increased the likelihood of death compared to having a local first progression (nodal: sub-HR: 3.13, 95% CI: 1.37,7.15; distant: sub-HR: 2.73, 95% CI: 1.28,5.81). The presence of a second progression regardless of the type, greatly influenced the melanoma-specific mortality compared to the absence of it, more specifically by about 6 times for the local (sub-HR: 5.78, 95% CI: 2.56,13.06), 10 times for the nodal (sub-HR: 9.89, 95% CI: 5.41,18.07), and 9 times for the distant ones (sub-HR: 8.67, 95% CI: 4.55,16.52). Furthermore, the presence of a distant third progression, in particular, increased by about 2.5 times the mortality risk compared to absence of it (sub-HR: 2.51, 95% CI: 1.29,4.89). The results were consistent when we also included the Bologna center and, hence, excluded the information on the third progression from the model (not reported).

## 4. Discussion

The colossal improvements in the detection methodology of CMs, which are fueling the debate about the “melanoma epidemic” [18], advocate for the fact that thin CMs are responsible for a substantial portion of melanoma’s incidence nowadays [11]. While thin CMs can metastasize, considerable efforts to define the subgroups of thin CMs at a higher risk for disease progression from a clinical and histopathological standpoint have been made so far [12,19,20]. The recent changes in the subcategorization of the T classification according to the 8th edition of the AJCC criteria have also raised a debate with respect to the optimal management of T1 CMs and the optimal follow-up of T1 melanoma patients [10,17]. We have, therefore, collected 205 thin CM cases with at least one progression and thereafter recorded the follow-up from five specialized centers in an attempt to describe the topographical and chronological sequences of thin CMs’ progressions, as well as define the natural history of the disease in order to identify possible distinct patterns and underlying mechanisms of its progression.

The greatest portion of the entire sample (61%) was surprisingly diagnosed with a distant first progression, namely, in solid organs or in a distant nodal basin. The distant site progression was more likely associated with the male sex, younger individuals, localization on the trunk, higher Breslow thickness, and an SSM histological subtype. A possible explanatory scenario for this paradoxical observation could be that the cancerous load of the primary thin CMs bypassed the regional lymphatic drainage basin and disseminated directly and hematogenously (what could be considered a skip phenomenon). This may be attributed either to its small quantity and/or its low immunogenic profile or to a clinically or histopathologically undetected or regressed regional nodal metastasis [21,22,23]. The mean time to progression was 3 years, thereby indicating that the progression process is either slow—an observation that has been reported before [11,24]—or that we only diagnose it on a late stage after it has surpassed intermediate progression steps. In fact, the risk of progression in stage Ia melanoma has been found to be approximately 1% per year [25] and the time to progression has been estimated to be 3.32 years (IQR: 1.72–6.14), with some patients’ first relapses at 17 years after diagnosis [11]. Taking into account the established management protocols of CM patients [17,26], there is an unmet need to discover new biomarkers for recognizing stage Ia patients at a higher risk for progression and, most critically, for distinguishing between fast relapsers and slow ones in order to individualize their monitoring. We also suggest considering the personal history of stage Ia melanoma, which is a key fact that all patients should be informed of and have discussions with their health care providers (nurses or doctors) to be considered in future health evaluations. Meanwhile, it is crucial to train patients in self-examination, including palpation, in order to identify affected locoregional lymph nodes or skin metastases and to encourage them to seek expert health care should any symptom appear.

Almost half of the sample with a first progression and recorded data (107/190, 56%) manifested a second one, which is an expected finding based on the underlying mechanisms of distant first metastasis, which occurred in the majority of the cases. The second progression developed after a mean interval of 0.82 years between the first and second progression. This shorter interval compared to the time to reach the first progression is a predictable finding, as tumors that have already metastasized, especially directly in distant locations, as was the case for most of our sample’s cases, have accumulated a mutational profile that is synonymous with a more aggressive biological behavior and have started to spread systematically at a microscopic level. It is worth nothing that the risk for a second progression increased for the first 3 years after the first progression. Thin CMs of the LMM histotype were at a lower risk for a second progression, which seems compatible with the natural history of this type of melanoma [27], whereas CMs with a local first progression were at a higher risk for a second one, possibly due to the dissemination of the residual, cancerous cells in the cutaneous territory of the primary tumor. Moreover, the Breslow thickness of the primary tumor was positively associated with a nodal second metastasis. Finally, the third progression occurred even earlier, in a mean time interval of 0.43 years after the second progression, without any association with demographic, clinical, or histopathological characteristics being identified.

A total of 50% (86/172) of the patients with follow-up records died due to melanoma-related causes. The mean time between the diagnosis of the first progression and death was 4 years. The melanoma-specific mortality was positively correlated with the presence of a nodal or distant first progression, the occurrence of a second progression—regardless of localization—and with a distant third progression. Naturally, our observations differ dramatically from the melanoma-specific survival in the cases of stage I thin CMs [28,29].

## 5. Conclusions

The up-to-date developed therapeutic strategies for efficiently targeting late-stage CM, such as adjuvant therapies, have significantly improved the prognosis of melanoma patients, including those diagnosed with a thin CM that has progressed. The observations of the study remarkably describe the natural course of thin CM progression, as most of the cases manifested progressions before 2018 and were not impacted by the introduction of novel therapies, which, according to existing guidelines, would have been infeasible. Consistent with other similar studies [30,31,32], they confirm that, despite thin CM’s excellent prognosis overall, there is a subgroup of the disease that metastasizes and adds to CM’s mortality [6], thereby highlighting the heterogeneity of thin CMs while putting the 1 mm Breslow thickness cutoff into question [12,33]. It is of the utmost importance to define epidemiologically, histopathologically, and molecularly the distinct subpopulation within the thin CMs that harbors a higher potential for metastasis and, therefore, significantly reduced survival with additional, appropriate studies. Until then, it is reasonable to consider the abandonment of the term “thin melanoma”, as it may falsely encourage attenuated vigilance among physicians and preoccupy the public with an altogether positive outcome. Furthermore, the observations generally underscore the need for the optimization of the management of patients with thin CM toward a more detailed and personalized approach based on the topographical and chronological steps of its progression.

## Figures and Tables

**Table 1:** Characteristics of the patients included in the study dataset (n = 204).

Characteristics	
Center n = 204, 0 missing	N(%)
Barcelona	36(17.6)
Badalona	18(8.8)
Valencia	34(16.7)
Turin	98(48.0)
Bologna	18(8.8)
Sex (n = 204, 0 missing)	
Male	100(49.0)
Female	104(51.0)
Age at diagnosis (median, IQR, in years, 0 missing)	52.5(40.0,64.9)
Year at diagnosis (median, IQR, in years, 0 missing) Year at latest follow-up or death (median, IQR, in years, 0 missing)	2001(1992,2007) 2012(2003,2018)
Body site (n = 204, 0 missing)	
Head/Neck	26(12.7)
Trunk	96(47.1)
Arms	27(13.2)
Legs	55(27.0)
Breslow score (median, IQR, 0 missing)	0.8(0.6,0.9)
Ulceration (n = 203, 1 missing)	
No	183(90.2)
Yes	20(9.8)
Histological subtype (n = 194, 10 missing)	
SSM	155(79.9)
LMM	14(7.2)
NM	13(6.7)
Sentinel lymph node biopsy (n = 204, 0 missing)	
No	147(72.1)
Yes	57(27.9)
Regional nodal	30(14.7)
Distant or subcutaneous	125(61.3)
Elapsed time between diagnosis and first progression (median, IQR, years, 1 missing)	3.10 (1.09,5.24)

**Table 2:** Univariate and multivariable analysis: subhazards ratio (sub-HR) of second progression estimated by Fine and Gray model (n = 190). Bold numbers correspond to statistically significant values.

Variable	Univariate Sub-HR	95% CI	Multivariable Sub-HR	95% CI
Sex				
Male	1.00	ref	1.00	ref
Female	0.90	0.62,1.31	0.83	0.54,1.27
Body site				
Head/Neck	0.98	0.56,1.71	1.50	0.69,3.26
Trunk	1.00	ref	1.00	ref
Arms	0.87	0.48,1.56	0.94	0.52,1.71
Legs	1.02	0.66,1.59	1.06	0.63,1.78
Breslow score (unit increase)	1.73	0.76,3.94	2.13	0.80,5.66
Ulceration				
No	1.00	ref	1.00	ref
Yes	1.40	0.76,2.58	1.48	0.82,2.70
Histological subtype				
SSM	1.00	ref	1.00	ref
LMM	0.53	0.26,1.06	0.32	**0.14,0.76**
NM	1.01	0.45,2.28	1.02	0.43,2.45
ALM	0.61	0.22,1.70	0.65	0.21,1.98
Sentinel lymph node				
No	1.00	ref	1.00	ref
Yes	0.84	0.54,1.31	0.79	0.50,1.25
Type of first progression				
Local	1.00	ref	1.00	ref
Nodal	0.87	0.49,1.55	0.85	0.45,1.58
Distant	0.77	0.52,1.15	0.67	0.42,1.05
Age at diagnosis (unit increase)	1.00	0.99,1.02	1.01	0.99,1.02
Elapsed time between diagnosis and first progression (years)				
[0–1)	1.00	ref	1.00	ref
[1–2)	1.47	0.74,2.89	1.26	0.60,2.65
[2–3)	1.80	0.89,3.64	1.72	0.83,3.53
3+	1.15	0.67,1.99	1.10	0.62,1.95

**Table 3:** Univariate and multivariable analysis among patients with a second progression: subhazards ratio (sub-HR) of third progression estimated by Fine and Gray model (n = 99).

Variable	Univariate Sub-HR	95% CI	Multivariable Sub-HR	95% CI
Sex				
Male	1.00	ref	1.00	ref
Female	1.19	0.68,2.08	1.04	0.48,2.25
Body site				
Head/Neck	1.25	0.53,2.92	1.20	0.41,3.48
Trunk	1.00	ref	1.00	ref
Arms	0.51	0.16,1.62	0.47	0.13,1.72
Legs	0.98	0.54,1.83	0.63	0.27,1.47
Breslow score (unit increase)	0.55	0.17,1.83	0.46	0.09,2.29
Ulceration				
No	1.00	ref	1.00	ref
Yes	0.37	0.12,1.13	0.32	0.08,1.23
Histological subtype				
SSM	1.00	ref	1.00	ref
LMM	0.59	0.17,2.05	0.34	0.09,1.33
NM	1.32	0.40,4.36	1.80	0.40,8.11
ALM	1.20	0.39,3.75	1.44	0.40,5.17
Sentinel lymph node				
No	1.00	ref	1.00	ref
Yes	0.92	0.49,1.72	1.37	0.67,2.81
Type of second progression				
Local	1.00	ref	1.00	ref
Nodal	0.61	0.33,1.15	0.52	0.23,1.18
Distant	1.02	0.53,1.95	1.09	0.44,2.65
Age at diagnosis (unit increase)	0.99	0.97,1.01	0.98	0.96,1.01
Elapsed time between diagnosis and second progression (years)				
[0–2)	1.00	ref	1.00	ref
[2–4)	0.93	0.33,2.64	0.78	0.25,2.49
[4–6)	1.24	0.46,3.37	1.11	0.31,4.03
6+	1.26	0.47,3.33	1.20	0.36,3.98

**Table 4:** Univariate and multivariable analysis: subhazards ratio (sub-HR) of mortality estimated by Fine and Gray model (n = 172). Bold numbers correspond to statistically significant values.

Variable	Univariate Sub-HR	95% CI	Multivariable Sub-HR	95% CI
Sex				
Male	1.00	ref	1.00	ref
Female	0.70	0.46,1.06	0.96	0.58,1.60
Body site				
Head/Neck	0.41	**0.18,0.91**	1.11	0.53,2.33
Trunk	1.00	ref	1.00	ref
Arms	0.47	**0.23,0.96**	0.52	0.23,1.21
Legs	0.44	**0.27,0.74**	0.59	0.29,1.19
Breslow score (unit increase)	2.90	1.00,8.43	2.46	0.60,10.10
Ulceration				
No	1.00	ref	1.00	ref
Yes	1.68	0.91,3.11	1.73	0.79,3.75
Histological subtype				
SSM	1.00	ref	1.00	ref
LMM	0.34	0.11,1.06	0.43	0.09,2.08
NM	1.11	0.50,2.44	1.11	0.34,3.61
ALM	1.44	0.54,3.83	2.18	0.58,8.10
Sentinel lymph node				
No	1.00	ref	1.00	ref
Yes	1.00	0.61,1.63	0.84	0.42,1.67
Type of first progression				
Local	1.00	ref	1.00	ref
Nodal	1.33	**1.69,8.47**	3.13	**1.37,7.15**
Distant	2.10	**3.40,19.81**	2.73	**1.28,5.81**
Type of second progression				
None	1.00	ref	1.00	ref
Local	3.93	**1.65,9.34**	5.78	**2.56,13.06**
Nodal	14.19	**8.28,24.31**	9.89	**5.41,18.07**
Distant	12.05	**6.48,22.38**	8.67	**4.55,16.52**
Type of third progression				
None	1.00	ref	1.00	ref
Local	0.85	0.13,5.68	0.68	0.19,2.43
Nodal	3.73	**2.53,5.49**	1.43	0.81,2.51
Distant	3.66	**2.01,6.68**	2.51	**1.29,4.89**
Age at diagnosis (unit increase)	1.00	0.99,1.02	1.01	0.99,1.03
Elapsed time between diagnosis and first progression (years)				
[0–1)	1.00	ref	1.00	ref
[1–2)	1.05	0.45,2.48	1.29	0.54,3.07
[2–3)	1.78	0.81,3.93	1.35	0.53,3.46
3+	1.29	0.70,2.38	1.34	0.61,2.91

## Data Availability

The data presented in this study are available upon request from the corresponding author. The data are not publicly available due to privacy restrictions. No public datasets were generated or analyzed during the current study.
